# Metformin suppresses hypoxia-induced stabilization of HIF-1α through reprogramming of oxygen metabolism in hepatocellular carcinoma

**DOI:** 10.18632/oncotarget.6418

**Published:** 2015-11-28

**Authors:** Xinke Zhou, Jitao Chen, Gao Yi, Min Deng, Hao Liu, Min Liang, Boyun Shi, Xin Fu, Yuqin Chen, Liangcai Chen, Zhimin He, Jian Wang, Jifang Liu

**Affiliations:** ^1^ Department of Respiratory Medicine and Cancer Center, The 5th Affiliated Hospital of Guangzhou Medical University, Guangzhou, PR China; ^2^ Cancer Hospital and Cancer Research Institute, Guangzhou Medical University, Guangzhou, PR China; ^3^ State Key Laboratory of Respiratory Disease, The 1st Affiliated Hospital of Guangzhou Medical University, Guangzhou, PR China

**Keywords:** HIF-1α, metformin, metabolic reprogramming, hypoxia, hepatocellular carcinoma

## Abstract

Overexpression of hypoxia-induced factor 1α (HIF-1α) has been shown to be involved in the development and progression of hepatocellular carcinoma (HCC). HIF-1α should therefore be a promising molecular target for the development of anti-HCC agents. Metformin, an established antidiabetic drug, has proved to also be effective in treating cancer although the precise underlying mechanisms of this activity are not fully elucidated. The aim of this study was to investigate the effects of metformin on the expression of HIF-1α and oxygen metabolism in HCC. The results showed that metformin inhibited hypoxia-induced HIF-1α accumulation and activation independent of AMP-activated protein kinase (AMPK). Moreover, this decrease in HIF-1α accumulation was accompanied by promotion of HIF-1α protein degradation. In addition, metformin significantly decreased oxygen consumption, ultimately leading to increased intracellular oxygen tension and decreased staining with the hypoxia marker pimonidazole. *In vivo* studies demonstrated that metformin delayed tumor growth and attenuated the expression of HIF-1α in HCC tumor xenografts. Together, these findings suggest that metformin decreases hypoxia-induced HIF-1α accumulation by actively suppressing mitochondrial oxygen consumption and enhancing cellular oxygenation ability, providing a fundamental mechanism of metformin activity against HCC.

## INTRODUCTION

Hepatocellular carcinoma (HCC) is one of the most prevalent fatal cancers worldwide and the second leading cause of death in China. Multiple environmental and genetic risk factors are associated with the high incidence of HCC development and progression and unsatisfactory mortality rate. Thus, a better understanding of the molecular mechanisms governing hepatocarcinogenesis and HCC progression will contribute to identification of new therapeutic strategies for this highly malignant tumor.

Increasing evidence suggests that intratumor hypoxia is a common phenomenon of solid tumors, including HCC, and an important microenvironmental factor influencing development of a malignant phenotype [1]. Hypoxia-inducible factor 1 (HIF-1), a heterodimer consisting of α and β subunits, is a key transcription factor regulating the cellular response to hypoxia [2]. HIF-1α has been found to be upregulated in a variety of human malignancies, leading to an increased capacity for metastasis and an unfavorable prognosis [3, 4]. Whereas HIF-1β is a constitutive nuclear protein, HIF-1α is tightly regulated according to oxygen availability through its protein stability. In the presence of abundant oxygen, HIF-1α is rapidly degraded by polyubiquitination via the Von-Hippel-Lindau tumor suppressor protein and subsequent proteasomal degradation [5]. Under hypoxic conditions, HIF-1α is stabilized and translocates to the nucleus, where it dimerizes with HIF-1β and activates the expression of a broad range of target genes including glucose transporter 1 (Glut1) and carbonic anhydrase IX (CAIX), facilitating tumorigenesis and cancer progression [6–8].

Metformin, a commonly prescribed biguanide, has been used as a first-line treatment for type II diabetes for over 50 years. It efficiently and safely lowers blood glucose levels without serious side effects [9, 10]. Recently, increasing lines of epidemiologic evidence have revealed that diabetic patients who are treated with metformin have reduced cancer incidence and mortality [11, 12]. In addition, a great number of *in vitro* and *in vivo* studies have revealed a direct action of metformin on many types of cancer cell, including HCC [13–15]. Metformin may therefore be a potential therapeutic agent for the treatment of HCC, although its mechanism of anticancer action remains unclear. Recently, several studies have demonstrated that metformin inhibits HIF-1α expression in patients with breast cancer [16] or hepatocellular carcinoma Bel-7402/5-fluorouracil (*Bel-Fu*) cells [17]. However, the mechanism of such regulation remains to be elucidated. Moreover, metformin has been found to activate AMP-activated protein kinase (AMPK), a major sensor of the energetic status of the cell [18], via a mechanism dependent on liver kinase B1 (LKB1) [19, 20]. The LKB1–AMPK–mammalian target of rapamycin (mTOR) pathway is a key moderator of HIF-1-targeted genes and HIF-1-mediated cellular metabolism [21]. Therefore, we questioned whether metformin, which is a known AMPK activator, regulates the expression of HIF-1α protein in HCC cells. The present study aims to explore the effect of metformin on HIF-1α expression and activation in HCC cells and xenografts.

## RESULTS

### Metformin represses hypoxia-induced accumulation of HIF-1α protein in hepatoma cells

**Figure 1:**
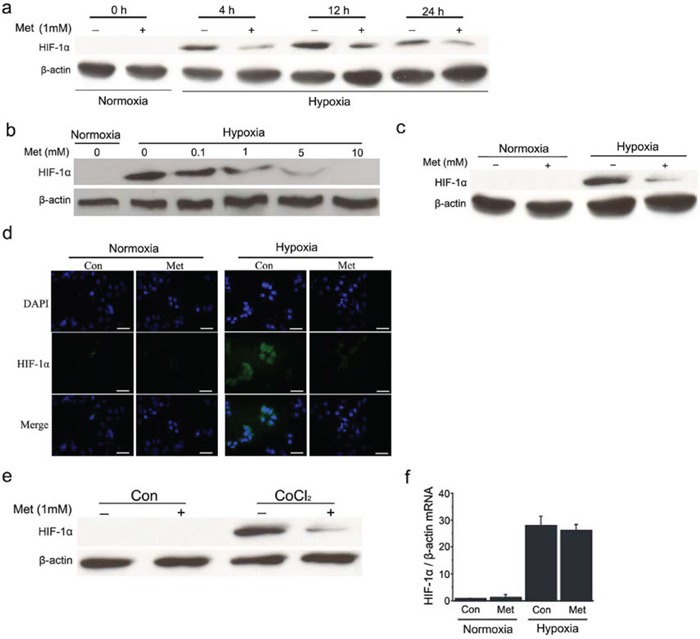
Metformin decreases HIF-1α protein levels in HCC cells **a.** and **b.** Western blot analysis of HIF-1α protein in HepG_2_ cells treated for different times or with various concentrations of metformin as indicated under normoxia or hypoxia. **c.** and **d.** Expression of HIF-1α protein was detected using western blot and immunofluorescence analysis, respectively, in Huh7 cells with or without metformin treatment under normoxic or hypoxic conditions. Scale bars: 50 μm. **e.** HepG_2_ cells were pretreated with 100 μmol/L CoCl_2_ for 3 h before incubation with 1 mmol/L metformin for 12 h and the protein expression of HIF-1α was assayed by western blotting. **f.** Relative expression of *HIF-1α* mRNA was examined by real-time RT-PCR in HepG_2_ cells in the presence or absence of metformin. β-actin was used as the internal loading control. Con, control; Met, metformin.

To examine the effect of metformin on HIF-1α protein content, time-course and dose-response experiments were conducted to determine alterations in HIF-1α protein in the presence of metformin. As expected, HIF-1α protein was undetectable in HepG_2_ cells under normoxia, but its expression markedly increased under hypoxia. In the presence of metformin, HIF-1α protein content was dramatically diminished and this decrease persisted as long as the drug was present for at least up to 24 h under hypoxic conditions (Figure 1a). Dose-response experiments demonstrated that metformin attenuated hypoxia-induced HIF-1α protein expression in HepG_2_ cells in a dose-dependent manner, with complete abrogation at 10 mmol/L (Figure 1b). Further confirming the effect of metformin, the accumulation of HIF-1α was also diminished in another HCC cell line, Huh7, by treatment with metformin at a concentration of 1 mmol/L (Figure 1c and 1d). In addition, CoCl_2_, a hypoxia-mimicking reagent, induced accumulation of HIF-1α and this increase could also be decreased by metformin (Figure 1e). In contrast, *HIF-1α* mRNA levels were not reduced by metformin under hypoxic conditions (Figure 1f), suggesting that metformin decreased HIF-1α protein expression via post-translational mechanisms.

### Metformin inhibits hypoxia-induced HIF-1α transactivation activity

**Figure 2:**
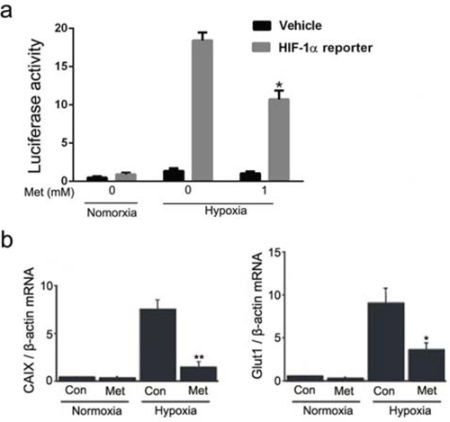
Effect of metformin on transactivation activity of HIF-1α in HepG2 cells **a.** Luciferase activity was measured in HepG_2_ cells that were transiently transfected with a HIF-1α reporter gene and then treated with 1 mmol/L metformin for 12 h under normoxic or hypoxic conditions. *P < 0.05 compared with cells transfected with HIF-1α reporter without metformin treatment in hypoxia. **b.** Real-time RT-PCR analyses of *CAIX* and *Glut1* mRNA expression in HepG_2_ cells with or without metformin treatment under normoxic and hypoxic conditions. The relative amounts of *CAIX* and *Glut1* mRNA were normalized to β-actin expression. Hypoxia significantly induced *CAIX* and *Glut-1* mRNA expression in HepG_2_ cells, and 1 mmol/L metformin inhibited the induction of these mRNAs in hypoxia. *, *P* < 0.05; **, *P* < 0.01 compared with control under hypoxic conditions. All experiments were performed three times. Data shown represent the means ± SD. Con, control; Met, metformin.

To further determine whether metformin-mediated inhibition of HIF-1α protein expression leads to functional suppression of HIF-1α, we measured the transcriptional activity of HIF-1α in HepG_2_ cells transiently transfected with HIF-1α reporter vector and pEGFP-C2. As shown in Figure 2a, hypoxic stress increased luciferase activity up to 17-fold compared with the normoxic condition, whereas metformin significantly inhibited hypoxia-induced luciferase activity by 52.9%. We next verified whether inhibition of HIF-1α alters the transcriptional activation of HIF-1α target genes. Pretreatment with 1 mmol/L metformin significantly reduced the hypoxic induction of specific HIF-1α downstream genes *CAIX* and *Glut1*, as measured by quantitative RT-PCR (Figure 2b). These results were confirmed in Huh7 cells, which also showed a significant decrease in HIF-1α transactivation activity after metformin treatment (Figure S1).

### Metformin suppresses hypoxia-induced HIF-1α expression independent of AMPK

**Figure 3:**
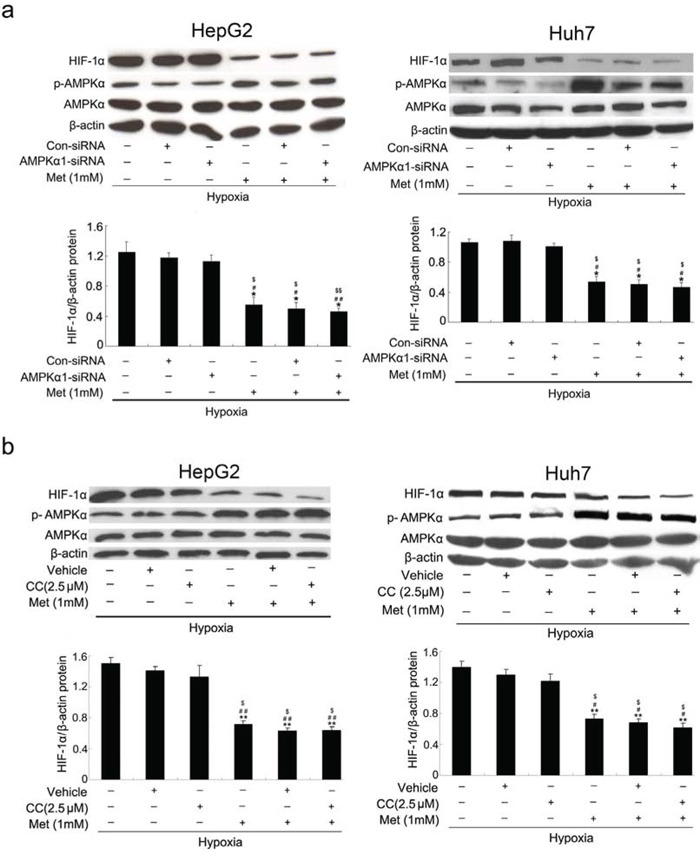
Metformin suppresses HIF-1α protein levels independent of AMPK **a.** and **b.** Western blot analyses of HIF-1α, AMPKα, and phosphorylated form of AMPKα (p-AMPKα). HepG_2_ and Huh7 cells were transiently transfected with AMPKα1 siRNA or treated with Compound C (CC; AMPK inhibitor), and then incubated with 1 mmol/L metformin for 12 h under hypoxic conditions before immunoblotting with specific antibodies as indicated. β-actin was used as the internal loading control. *, *P* < 0.05; **, *P* < 0.01 vs. untreated cells; #, *P* < 0.05; ##, *P* < 0.01 vs. control siRNA-transfected or vehicle-treated cells; $, *P* < 0.05; $$, *P* < 0.01 vs. AMPKα1-siRNA or CC-treated cells. Results from three independent experiments are shown.

Numerous reports have shown that metformin inhibits tumorigenesis in a manner dependent on AMPK [22, 23]. To investigate the effect of AMPK on the inhibitory activity of metformin on hypoxia-induced HIF-1α protein accumulation, we took a genetic or chemical approach to inhibit AMPK activity under hypoxic conditions. As shown in Figure 3, decreasing the level of AMPKα protein using specific siRNA or AMPK inhibitor did not affect hypoxia-induced HIF-1α expression and failed to restore the inhibitory effects of metformin on hypoxia-induced HIF-1α expression in both HepG_2_ and Huh7 cell lines, implying that metformin inhibited hypoxia-induced HIF-1α protein expression independent of AMPK.

### Metformin promotes protein degradation of HIF-1α

**Figure 4:**
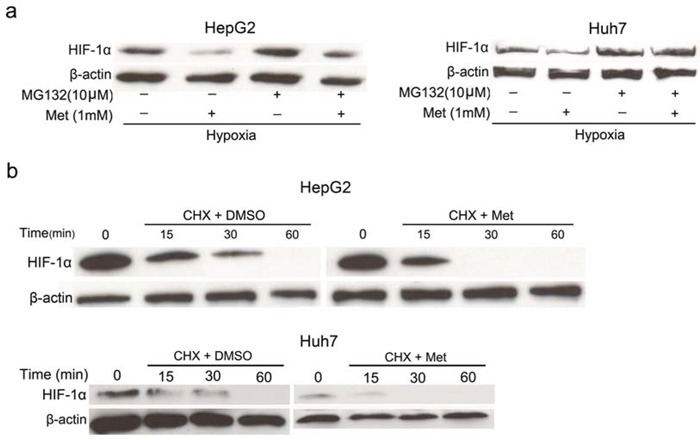
Effects of metformin on protein stability of HIF-1α **a.** The proteasomal inhibitor MG-132 (10 μmol/L) rescued the inhibitory effect of 1 mmol/L metformin on HIF-1α protein accumulation in hypoxia. **b.** HepG_2_ and Huh7 cells were cultured in hypoxia for 4 h, and then treated with CHX (4 μg/ml; a translational inhibitor) and metformin for 0, 15, 30, and 60 min in normoxia. The results indicated that metformin speeds up the degradation of HIF-1α protein.

In general, the accumulation of HIF-1α is dependent on the balance between its protein synthesis and degradation. HIF-1α is degraded mainly through the ubiquitin-proteasome pathway [24, 25]. To investigate the involvement of proteasomal degradation in metformin-treated cells, we treated HepG_2_ and Huh7 cells with MG132 (a proteasomal inhibitor) for 4 h, followed by co-incubation with metformin for 4 h in hypoxic conditions. As shown in Figure 4a, MG-132 restored the inhibitory effect of metformin on hypoxia-induced HIF-1α accumulation. To further confirm the involvement of metformin in the degradation of HIF-1α, the half-life of HIF-1α was examined in normoxia with or without metformin treatment. HepG_2_ and Huh7 cells were pretreated in hypoxia for 4 h and then co-incubated with cycloheximide (CHX, a global protein synthesis inhibitor) and metformin for 0, 15, 30, and 60 min under normoxic conditions. These results demonstrated that the protein degradation rate of HIF-1α was not significantly altered in metformin-treated and control cells at 15 min whereas HIF-1α protein was completely degraded in the metformin-treated cells at 30 min, in sharp contrast to HIF-1α in the control cells (Figure 4b). Collectively, these data show that the inhibitory effect of metformin on HIF-1α accumulation is mediated by an increase in protein degradation.

### Decreased oxygen consumption correlates with degradation of HIF-1α protein in metformin-treated cells

**Figure 5:**
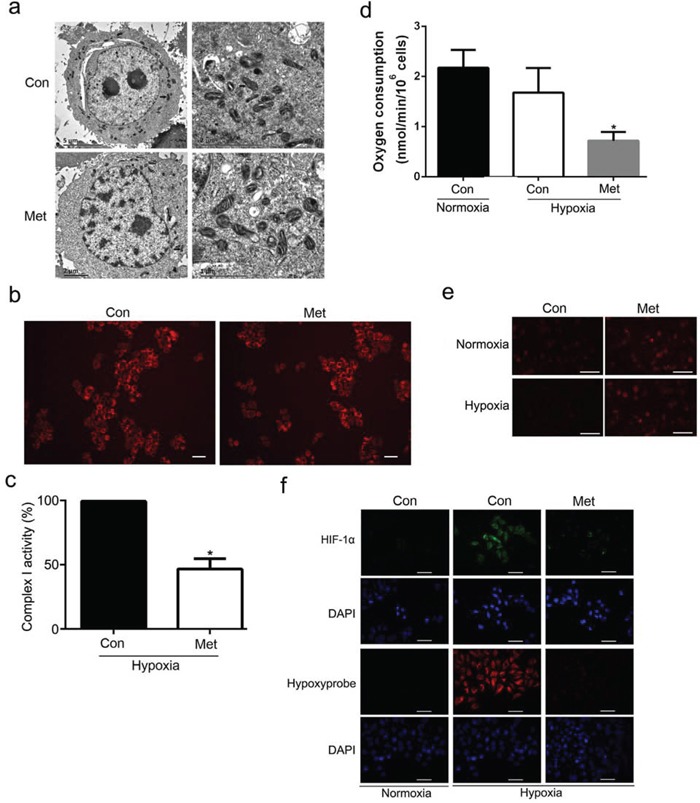
Effect of metformin on mitochondrial biogenesis and oxygen metabolism **a.** Representative transmission electron microscopy images of mitochondria in HepG_2_ cells. **b.** Fluorescence microscopy analysis of mitochondrial mass in HepG_2_ cells after staining with mitotracker red. **c.** Changes in mitochondrial complex I activity in response to metformin treatment in hypoxia. The control value was set at 100%. *P < 0.05 versus the control. **d.** Metformin inhibited mitochondrial respiratory functions of HepG_2_ in normoxia and hypoxia. Cells were treated with 1 mmol/L metformin in normoxia or hypoxia for 4 h and then resuspended in normoxic medium. Oxygen consumption was measured in a sealed chamber using a Clark-type electrode. *P < 0.05 compared with control in hypoxia. **e.** Effect of metformin on ROS production measured with DHE. HepG_2_ cells were treated with 1 mmol/L metformin for 4 h under normoxic or hypoxic conditions and then incubated with 2.5 μmol/L DHE and viewed under a fluorescence microscope. **f.** Detection of hypoxic state and HIF-1α expression in HepG_2_ cells by immunocytochemical analysis. Nuclei were stained with DAPI. Scale bars: 50 μm. All results are representative of three independent experiments. Values are given as the mean ± SD. Con, control; Met, metformin.

In earlier work, we found that a low dose of metformin decreased mitochondrial respiratory chain complex I activity, leading to depletion of intracellular ATP levels [26]. To address the underlying mechanisms responsible for the metformin-mediated increase in HIF-1α protein degradation, we investigated the effect of metformin on mitochondrial biogenesis, mitochondrial respiratory chain complex I activity, oxygen consumption, and reactive oxygen species (ROS) production. As shown in Figure 5a and 5b, metformin had no significant effect on mitochondrial morphology and number in HepG_2_ cells compared with controls under hypoxic conditions. In accordance with our previous study [26], metformin significantly inhibited complex I activity in hypoxia (Figure 5c). Oxygen consumption was also measured in HepG_2_ cells treated with 1 mmol/L metformin under normoxic and hypoxic conditions for 4 h. As shown in Figure 5d, hypoxia decreased oxygen consumption to 71.7% that of the control in normoxia, which was similar to the results of the previous study [27]. Of note, metformin significantly blunted oxygen consumption compared with control cells under hypoxic conditions. To further explore whether ROS production is implicated in the inhibitory effects of metformin on HIF-1α expression, we examined DHE staining, which produces a red fluorescence in a typically nuclear localization when oxidized to ethidium bromide by O_2_, in HepG_2_ cells. As shown in Figure 5e, hypoxia decreased DHE-associated fluorescence compared with normoxic conditions. However, metformin increased DHE staining independent of oxygen tension, as reported by Anedda et al. [28], suggesting that intracellular ROS production was not involved in the inhibitory effect of metformin on HIF-1α expression. Intriguingly, treatment of HepG_2_ cells with metformin significantly attenuated the intensity of pimonidazole staining (a hypoxia-sensitive marker), suggesting that metformin rescues the hypoxic state and increases intracellular oxygen tension under hypoxic conditions (Figure 5f). Additionally, hypoxia appeared to induce nuclear expression of HIF-1α compared with normoxic conditions, and metformin inhibited hypoxia-induced HIF-1α nuclear staining (Figure 5f). Similarly, cellular oxygen consumption was significantly lower in metformin-treated Huh7 cells than in control cells (Figure S2). Taken together, these results indicate that downregulation of cellular oxygen consumption via the induction of mitochondrial respiration dysfunction by metformin at least partially contributes to the O_2_-dependent degradation of HIF-1α.

### Metformin decreases HIF-1α expression in HCC xenografts

**Figure 6:**
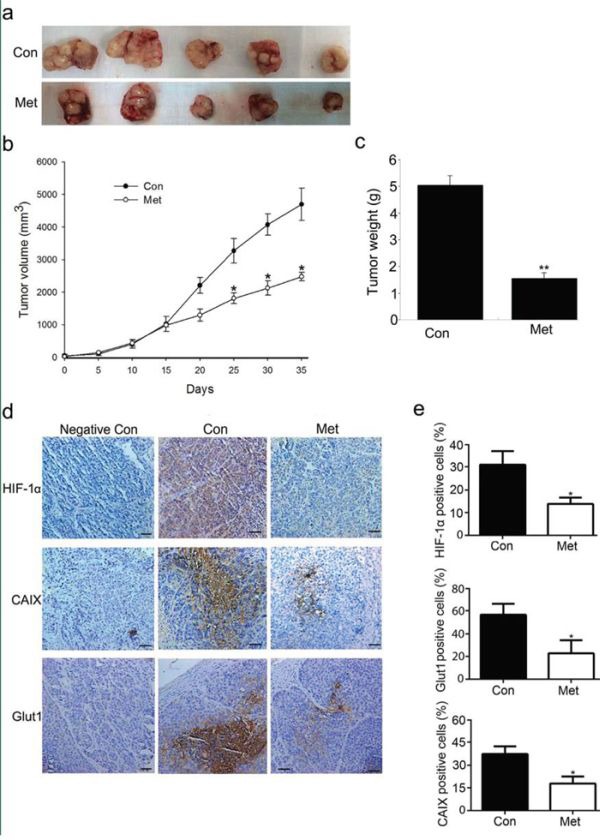
Metformin administration inhibits HCC xenograft tumor growth **a.** Representative image of tumors dissected from vehicle-treated control mice and metformin-treated mice at 35 d. **b.** Tumor growth in vehicle-treated control mice and metformin-treated mice. After tumor formation, mice were treated daily with intraperitoneal injection of metformin (250 mg/kg). **c.** Quantification of tumor weight 35 days post-tumor formation. Metformin significantly delayed tumor growth. **d.** Expression of HIF-1α, Glut1, and CAIX in tumor tissues was assessed by immunostaining and statistical graphs **e.** are shown. Scale bars: 50 μm. Data are represented as means ± SD of each group. *P < 0.05, **P < 0.01 compared with control. Con, control; Met, metformin.

To further investigate the effect of metformin on the expression of HIF-1α *in vivo*, we treated nude mice bearing HCC xenografts with 250 mg/kg body weight of metformin for 35 days. As expected, treatment of subcutaneously implanted HepG_2_ cell tumor xenografts with metformin obviously prevented tumor growth and proliferation, as measured by Ki67 expression (Figure 6 and Figure S3). As shown in Figure 6a, mice treated with metformin exhibited smaller tumors in the HepG_2_ xenograft compared with vehicle-treated control mice. Measurement of tumors every 5 d showed that tumor growth was dramatically reduced in metformin-treated mice (Figure 6b). In line with these results, tumor weight at the end of the study was significantly diminished by metformin treatment (Figure 6c). A remarkable reduction in the protein levels of HIF-1α, Glut1, and CAIX was also observed in immunohistochemical analysis of sections of tumor tissue from mice treated with metformin (Figure 6d and 6e). These results confirmed that downregulation of HIF-1α by metformin significantly contributes to the inhibition of tumor growth in HCC xenografts.

## DISCUSSION

In this study, we showed that metformin decreases hypoxia-induced HIF-1α protein content by promoting protein degradation rather than through reduction of protein synthesis or mRNA transcription. Suppression of mitochondrial oxygen consumption by metformin was found to be involved in the degradation of HIF-1α. Furthermore, metformin decreased the expression of HIF-1α in HCC xenografts. These results reveal a mechanism by which metformin downregulates HIF-1α in HCC cells.

Like other solid tumors, HCC tumors have large hypoxic regions and hypoxia is an important microenvironmental factor in promoting HCC progression and metastasis. Adaptation of tumor cells to hypoxia is mainly regulated by HIF-1α, which has been reported to be upregulated in HCC [1]. Interestingly, Ling et al. [17] found that metformin treatment led to decreased HIF-1α expression in *Bel-Fu* cells, which was consistent with the previous observation that metformin therapy reduced serum levels of HIF-1α in patients with breast cancer [16]. Suppression of HIF-1α expression would open a new perspective for HCC therapy, but little is known about the mechanism of action of HIF-1α. A recent study demonstrated that metformin improves tumor oxygenation and radiotherapy outcomes [29]. Our data are consistent with this report and expand these findings by suggesting a mechanism by which metformin exerts its antitumor effect.

The currently proposed molecular mechanism for the anticancer action of metformin is primarily associated with inhibition of the mTOR pathway, dependent on or independent of AMPK activation. Significant inhibition of mTOR, and an increase in phosphorylated AMPK, was observed in pancreatic tissue of mice fed with metformin [30]. However, Memmott *et al*. demonstrated that metformin inhibited induction of the mTOR pathway in the absence of AMPK in mice lung cancer cells [31]. Sahra *et al*. similarly reported that metformin inhibited prostate cancer cell proliferation independent of AMPK activation [32]. Consistent with these observations, our present study showed that metformin inhibits hypoxia-induced HIF-1α protein accumulation independent of AMPK activation.

Intracellular HIF-1α protein content could be regulated at the level of protein translation or stability. In the present study, hypoxia-dependent HIF-1α stabilization was observed in the presence of the proteasome inhibitor MG-132, which suppresses the degradation of HIF-1α. In contrast, addition of cycloheximide to inhibit protein synthesis led to a much slower decline in HIF-1α, indicating that metformin does not inhibit HIF-1α synthesis but rather stimulates its degradation. The protein stability of HIF-1α is modulated by environmental oxygen concentration or intracellular ROS level [33, 34]. Although previous studies showed that mitochondria-generated ROS promote protein stability of HIF-1α [35, 36], in our study the protein stability of HIF-1α was not obviously changed by ROS, as measured by DHE staining. Interestingly, metformin efficiently reduces mitochondrial respiration, in accordance with a previous study showing that metformin impairs complex I of the respiratory chain leading to a marked decrease in respiration and tumorigenesis in colon cancer [37]. These data implied that a decrease in mitochondrial oxygen consumption of hepatoma cells in the presence of metformin involves the destabilization of hypoxia-induced HIF-1α.

In summary, metformin facilitates protein degradation of HIF-1α in HCC via suppression of mitochondrial oxygen consumption. These findings expand our understanding of the mechanism underlying the anticancer activity of metformin, and may help to optimize the treatment of HCC.

## MATERIALS AND METHODS

### Cell culture

Two human hepatoma cell lines (HepG_2_ and Huh7) obtained from the Cell Bank of Shanghai Institutes of Biological Sciences, Chinese Academy of Sciences were cultured in Dulbecco's modified Eagle medium (DMEM) supplemented with 10% fetal bovine serum (Invitrogen, Carlsbad, CA, USA) and 1% penicillin-streptomycin (Sigma, St Louis, MO, USA). Cell lines were maintained in a humidified atmosphere of 95% air and 5% CO_2_ at 37°C. Hypoxic culture conditions were achieved with a hypoxia incubator containing a gas mixture of 94% N_2_, 5% CO_2_, and 1% O_2_.

### Reagents and antibodies

Metformin (1,1 dimethylbiguanide-hydrochloride), dimethyl sulfoxide (DMSO), MG132, cobalt chloride (CoCl_2_), Compound C (CC), cycloheximide (CHX) and oxidative fluorescent dihydroethidium (DHE) were obtained from Sigma-Aldrich. A pimonidazole hydrochloride kit was purchased from Hypoxyprobe Inc. (Burlington, MA, USA). The primary antibodies against AMPKα, phospho-AMPK(Thr172), CAIX, Glut1, Ki67 and β-actin were obtained from Cell Signaling Technology (Beverly, MA, USA), and HIF-1α antibody was from Abcam Company (Cambridge, MA, USA).

### Western blotting

Aliquots of cell lysate containing 30–50 μg of protein from cells incubated under normoxic or hypoxic conditions were resolved by 10% SDS-PAGE and electrophoretically transferred onto PVDF membranes (Millipore Corporation, Billerica, MA, USA) in a transfer buffer. The membrane was washed and incubated with specific primary antibodies of interest followed by appropriate secondary antibodies, and visualized with enhanced chemiluminescence reagent (Thermo Scientific, Rockford, IL, USA).

### Quantitative real-time RT-PCR

Total RNA extraction was conducted using Trizol reagent (Invitrogen). First-strand cDNA synthesis was performed using a RevertAid First Strand cDNA synthesis kit (Thermo Scientific, MA, USA) following the manufacturer's protocol. Each cDNA sample was analyzed for gene expression by quantitative real-time PCR with an ABI 7500 Sequence Detector (Applied Biosystems, Foster City, CA, USA) using the SYBR Green Universal PCR Master Mix (Applied Biosystems). Specific primers for HIF-1α (forward 5′-AGTGTACCCTAACTAGCCG-3′, reverse 5′-CACAAATCAGCACCAAGC-3′), CAIX (forward 5′-TGTGCTCCTGGTTCTGTTCT-3′, reverse 5′-GCTCCTCGGGTGTCTTGT-3′), Glut1 (forward 5′-CGCCTTTGCCAGAGTTGA-3′, reverse 5′-TTCTTCCAAGCGAGACAGC-3′), and β-actin (forward 5′-GGCATGGGTCAGAAGGATT-3′, reverse 5′-CTTCTACAATGAGCTGCGTGTG-3′) were used in this study. The PCR reaction was performed at 95°C for 30 s, followed by 40 cycles at 95°C for 5 s and 58°C for 30 s. For each sample, the relative quantity of product was calculated using the 2^−ΔΔC(T)^ method.

### Oxygen consumption measurements

Cells were incubated under normoxic (21% O_2_) or hypoxic (1% O_2_) conditions and then treated with or without drugs for 4 h. The average intracellular oxygen consumption rate was measured in a sealed chamber using a Clark-type electrode according to the manufacturer's instructions.

### Transfection with siRNA

Silencing of *AMPKα1* gene expression was achieved by the siRNA strategy as described previously [38]. At 48 h after transfection, HepG_2_ and Huh7 cells were treated with 1 mmol/L metformin and subsequently incubated in hypoxia for 4 h. Protein levels were detected by western blot analysis.

### Luciferase assay

Cells (2 × 10^5^) were seeded in a 6-well plate and incubated overnight. Cells were transfected with 0.5 μg HIF-1α luciferase reporter vector designed to measure intracellular HIF-1α (Panomics, CA, USA) and 0.5 μg p-EGFP-C2 using lipofectamine 2000 (Invitrogen) according to the manufacturer's protocol. At 24 h after co-transfection, cells were treated with metformin and incubated under hypoxia for 12 h. Luciferase was measured using the luciferase assay system (Promega, Madison, MI, USA). pEGFP-C2 vector was used for normalization of transfection efficiency, and relative luciferase activity (defined as reporter activity) was calculated as the ratio of luciferase/EGFP activity.

### Determination of mitochondrial complex I activity

Complex I activity was determined using the Dipstick Assay kit (MitoSciences, Eugene, OR, USA) according to the manufacturer's protocols, as previously described [26].

### Imaging of reactive oxygen species

Dihydroethidium (DHE) was used to evaluate the intracellular production of superoxide. Briefly, HepG_2_ cells were cultured in the presence or absence of 1 mmol/L metformin for 4 h under normoxic and hypoxic conditions, washed with DMEM lacking serum and phenol red, and incubated with 2.5 μmol/L DHE. After incubation for 30 min in the dark, the cells were washed with cold PBS and examined by fluorescence microscopy.

### Detection of cellular hypoxia

Cellular hypoxia was detected by addition of pimonidazole hydrochloride, a marker of hypoxia that binds to cells according to oxygen pressure (pO_2_) levels. HepG_2_ cells treated with 1 mmol/L metformin were exposed to hypoxia for 4 h and then subjected to pimonidazole staining according to the manufacturer's instructions.

### *In vivo* study

All animal procedures were approved by the Animal Experimentation Ethics Committee of Guangzhou Medical University. Male nude mice (BALB/c, 4–6 weeks old) were purchased from the Laboratory Animal Services Center of Guangdong Province. To obtain HCC xenografts, 5 × 10^6^ cells were injected subcutaneously into the right flank of each mouse. At 12 days after cell implantation, the mice were randomly divided into groups (six mice/group). Mice in the experimental group were intraperitoneally injected with metformin at a dose of 250 mg/kg body weight per day whereas the control group received an equal volume of normal saline. Tumor volume was calculated every 5 days using the equation: [length×(width)^2^/2]. The mice were sacrificed after 35 days and the tumors were weighed and then fixed in 10% formalin for immunohistochemical staining.

### Immunohistochemistry

Paraffin-embedded, formalin-fixed tissues were immunostained for HIF-1α, CAIX, and Glut1 protein using standard immunohistochemistry procedures according to the manufacturers' instruction. Immunostained slides were evaluated independently by two pathologists in a double-blind manner. Negative controls were prepared in the same way but without primary antibodies.

### Statistical analyses

The SPSS 16.0 and Sigmaplot 10.0 programs were used for general statistical analysis. Experimental data were expressed as the mean ± SD. Differences between groups were assessed by Student *t* test for comparison of only two groups, or using one-way analysis of variance (ANOVA) for more than two groups. All tests performed were two-tailed. *P* < 0.05 was considered statistically significant.

## SUPPLEMENTARY FIGURES


