# Supplementary material for: Metformin suppresses hypoxia-induced stabilization of HIF-1α through reprogramming of oxygen metabolism in hepatocellular carcinoma
Source: Oncotarget. 2015 Nov 28;7(1):873–84. doi: 10.18632/oncotarget.6418 (PMC4808039; doi:10.18632/oncotarget.6418)
Supplement: Supplementary file 1 [file oncotarget-07-0873-s001.pdf]

SUPPLEMENTARY FIGURES

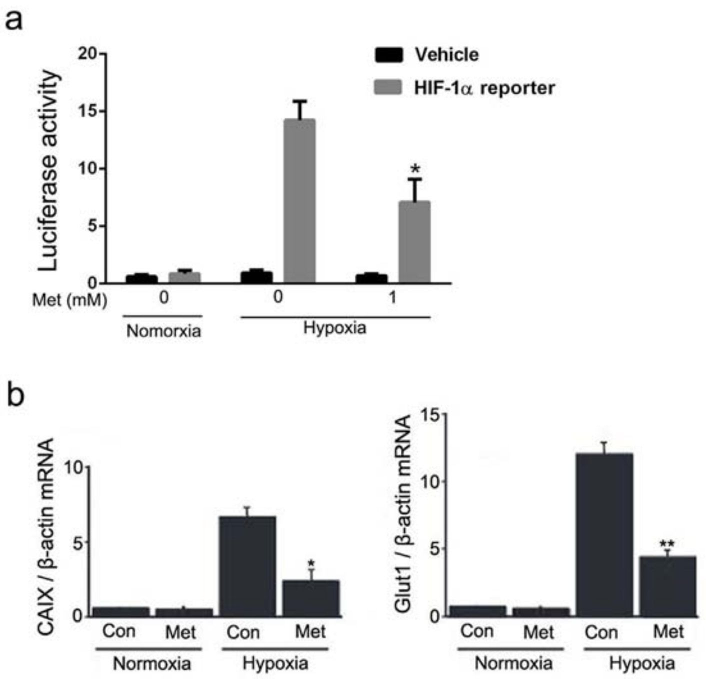

Supplementary Figure S1: Effect of metformin on transactivation activity of HIF-1α in Huh7 cells. a. Luciferase activity was measured in Huh7 cells that were transiently transfected with a HIF-1α reporter gene and then treated with 1 mmol/L metformin for 12 h under normoxic or hypoxic conditions. **P* < 0.05 compared with HIF-1α reporter transfected cells without metformin treatment in hypoxia. **b.** Real-time RT-PCR analyses of *CAIX* and *Glut1* mRNA expression in Huh7 cells with or without metformin treatment under normoxic and hypoxic conditions. The relative amounts of *CAIX* and *Glut1* mRNA were normalized to β-actin expression. **P* < 0.05, ***P* < 0.01 compared with control under hypoxic conditions. All experiments were performed three times. Data shown represent the means ± SD. Con, control; Met, metformin.

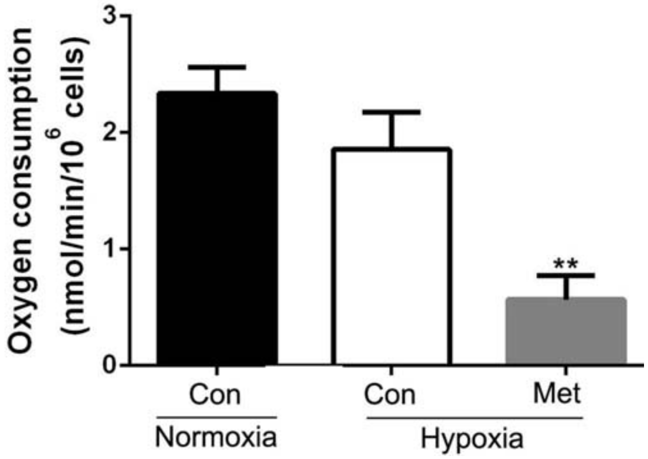

Supplementary Figure S2: Effect of metformin on oxygen consumption in Huh7 cells. Cells treated with 1 mmol/L metformin were incubated in normoxia or hypoxia for 4 h and then resuspended in normoxic medium. Oxygen consumption was measured in a sealed chamber using a Clark-type electrode. ***P* < 0.01 compared with control in hypoxia. Con, control; Met, metformin.

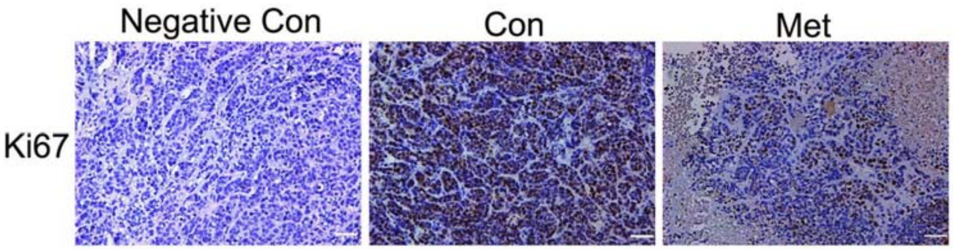

Supplementary Figure S3: Immunohistochemical detection of Ki67 in tumor tissue sections. Representative Ki67-stained sections of tumors grown in vehicle-treated mice and metformin-treated mice. Treatment with 1 mmol/L metformin suppressed tumor cell proliferation. Scale bars: 50 µm. Con, control; Met, metformin.
